# Treatment of Rheumatoid Arthritis with Marine and Botanical Oils: Influence on Serum Lipids

**DOI:** 10.1155/2011/827286

**Published:** 2011-10-09

**Authors:** Barbara C. Olendzki, Katherine Leung, Susan Van Buskirk, George Reed, Robert B. Zurier

**Affiliations:** ^1^Center for Integrative Nutrition, Division of Preventive and Behavioral Medicine, University of Massachusetts Medical School, Worcester, MA 01655, USA; ^2^Division of Preventive and Behavioral Medicine, University of Massachusetts Medical School, Worcester, MA 01655, USA; ^3^Department of Family Medicine and Community Health, University of Massachusetts Medical School, Worcester, MA 01655, USA; ^4^Division of Rheumatology, University of Massachusetts Medical School, Worcester, MA 01655, USA

## Abstract

The gap in mortality between patients with rheumatoid arthritis (RA) and the general population (1.5–3.0 fold risk) is increasing. This disparity is attributable mainly to cardiovascular disease (CVD), as the CVD risk is comparable to patients with diabetes mellitus. The purpose of this study is to determine whether borage seed oil rich in gamma-linolenic acid, fish oil rich in eicosapentaenoic acid (EPA) and docosahexaenoic acid (DHA), or the combination of both oils are useful treatments for dyslipidemia in patients with RA. We randomized patients into a double blind, 18 month trial. Mixed effects models were used to compare trends over time in serum lipids. No significant differences were observed between the three groups: All three treatment groups exhibited similar meaningful improvement in the lipid profile at 9 and 18 months. When all groups were combined, these treatments significantly reduced total and LDL-cholesterol and triglycerides, increased HDL-cholesterol, and improved the atherogenic index. All improvements observed at 9 months persisted at 18 months (*P* < 0.001 verses baseline). *Conclusion*. Marine and botanical oils may be useful treatment for rheumatoid arthritis patients who are at increased risk for cardiovascular disease compared to the general population.

## 1. Introduction

Over the past 30 years, substantial progress has been made in the medical and surgical management of patients with rheumatoid arthritis (RA). Despite this progress, there is an increasing gap in mortality between patients with RA (1.5–3.0 fold risk) and the general population. The disparity is mainly attributable to cardiovascular disease (CVD) [1] as the CVD risk is comparable to patients with diabetes mellitus [2, 3]. Although the reasons for this gap are not entirely clear, the traditional risk of abnormalities in lipid profiles [4] appears to be enhanced by a chronic increase in inflammatory cytokines [5], resulting in accelerated atherosclerosis. In fact, the elevated risk of cardiovascular disease for patients with RA indicates that atherosclerosis may in fact begin at lower thresholds of lipid dysfunction and inflammation than those in the general population, making the lipid profile and other risk factors of particular concern for patients with RA [6]. In a prospective study, [7] atherogenic lipid profiles were considerably worse in people who later met criteria for RA (as much as 10 years later) than those in matched controls. Although recent advances in the treatment of RA, especially with agents designed to block the actions of tumor necrosis factor alpha (TNF*α*), have improved the course of the disease [8] and endothelial function [9], results of studies of their influence on circulating lipids are mixed, and adequate evidence for or against such benefit is not available [10–12]. Due to the lack of supporting data, patients with RA, many of whom are limited in their ability to exercise to improve the lipid profile and the risk of CVD [13], have an urgent need for other treatments to control their dyslipidemia. 

It is clear that an increased intake of polyunsaturated fatty acids can improve their lipid profile [14]. Abundant experimental evidence supports the view that prostaglandins, thromboxanes, and leukotrienes (collectively termed eicosanoids), derived from polyunsaturated fatty acids, and participate in development and regulation of immunological and inflammatory responses [15–18]. The fatty acids themselves, by virtue of their incorporation into cell membranes and signal transduction elements, also have effects on cells involved in inflammation and immune responses that are independent of eicosanoids [18, 19]. A disease such as RA, characterized by abnormal immune responses, persistent inflammation, and joint tissue injury [20], may, therefore, be amenable to control by treatment with oils rich in specific polyunsaturated fatty acids.

**Figure 1:**
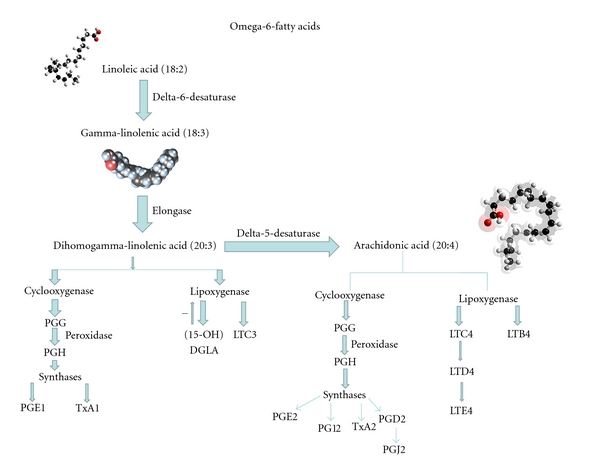


**Figure 2:**
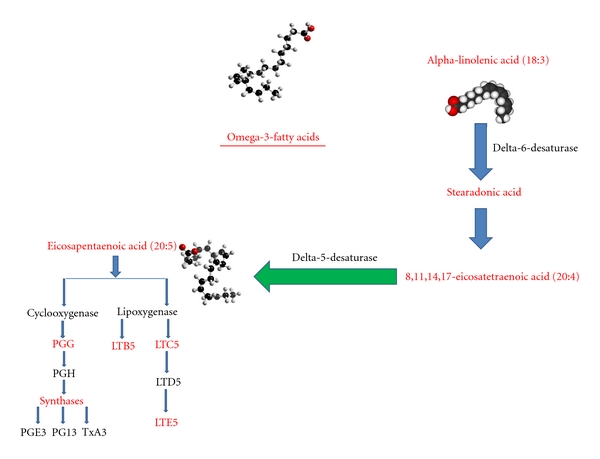


Gamma-linolenic acid (GLA: 18:3 omega 6, see Figure 1) is an essential fatty acid found in certain plant seed oils, including borage seed oil. GLA is metabolized to dihomogamma-linolenic acid (DGLA; 20:3 omega 6), the immediate precursor of prostaglandin E_1_ (PGE_1_), an eicosanoid with anti-inflammatory and immunoregulatory properties [18]. In addition, GLA cannot be converted to inflammatory leucotrienes by 5-lipoxygenase. Instead, it is converted to 15-hydroxy-DGLA which has the additional virtue of suppressing 5-lipoxygenase activity [21]. GLA and DGLA also modulate immune responses in an eicosanoid-independent manner by acting directly on T lymphocytes [18] and GLA suppresses acute and chronic inflammation, including arthritis, in animal models [18]. In addition, fish oil, rich in eicosapentaenoic acid (EPA; 20:5 omega 3, see Figure 2) and docosahexanoic acid (DHA; 22:6 w-3), suppresses formation of the inflammatory eicosanoids PGE_2_, thromboxane A_2_ (TXA_2_), and leucotriene B_4_ (LTB_4_). Randomized, placebo controlled double blind trials indicated that fish oil treatment of patients with RA result in clinical improvement, and those that monitored NSAID use suggest that fish oil treatment has an NSAID sparing effect [22].

A combination of EPA- and GLA-enriched oils exhibits synergy in reduction of synovitis in animal models [23], and administration of black currant seed oil, which contains the n-3 fatty acid alpha-linolenic acid (which is converted to EPA) and the n-6 GLA, suppresses active synovitis in patients with RA [24]. Taken together, these studies suggest that both EPA and GLA are beneficial therapies for patients with RA. With this knowledge, we carried out an 18-month, multi-center, randomized, double-blind, phase 3 trial of borage seed oil, fish oil, and a combination of both oils in patients with RA and active synovitis, to determine whether the combination of oils is superior to either oil used alone for the treatment of RA. Clinical outcomes of that study will be presented elsewhere. The object of the study presented here is to assess the influence of marine and botanical oils on serum lipids in patients with RA. 

## 2. Methods and Materials

The study was an 18-month randomized double-blind comparison of borage oil, fish oil, or a combination of both oils in RA patients with active joint inflammation. Patients received 3.5 gm omega-3 fatty acids daily in a 2.1 gm EPA/1.4 gm DHA ratio (7 fish oil and 6 sunflower oil capsules daily), 1.8 gm/d GLA (6 borage oil and 7 sunflower oil capsules/d), or 7 fish oil and 6 borage oil capsules daily (combination therapy). All capsules were identical in appearance and color and were purchased from the manufacturer, Bioriginal Food and Service Corp, Saskatoon, Canada, who shipped the capsules in opaque plastic bottles to the University of Massachusetts University Hospital pharmacy, from whence they were distributed to participating centers. Capsules were taken in 2 or 3 divided doses with meals.

 The protocol was reviewed and approved initially by the Committee for the Protection of Human Subjects at the University of Massachusetts Medical School and the Food and Drug Administration. Subsequent approvals were obtained from the Review Boards at the University of Alabama, Geisenger Clinic, Fallon Health Care, and the New England IRB. Written informed consent was obtained from each patient.

Patients were eligible to participate in the study if they had RA according to the 1987 criteria of the American Rheumatism Association [25], were in functional class I, II or III according to the revised criteria of the American College of Rheumatology [26], and were between the ages of 18 and 85. Patients were on a stable dose of drugs for RA for at least 2 months before the screening visit and a total duration of therapy of at least 6 months. Doses of nonsteroidal anti-inflammatory drugs (NSAID) and/or prednisone (<10 mg/d) were stable for at least 1 month before screening.

Patients were ineligible for the study if they had been treated with any investigational drug within 1 month of entry. If a patient was taking a fish oil supplement, the dose was stable and ≤2000 mg/d for 2 months before screening. If a patient was taking a borage oil supplement, the dose was stable and ≤2000 mg/d for 2 months before screening. An AST, ALT, or creatinine >1.5 times the upper limit of normal or a total bilirubin >1.8 mg/dL excluded patients from the trial. Patients were instructed to maintain their typical diet.

The lipid profile was assessed at baseline, 9, and 18 months. Diet was assessed by 24-hour dietary assessment calls (24-HR), performed at baseline and 18 months. Both laboratory evaluation and 24-HR were obtained in most patients who dropped out of the study before 18 months and after 3 months. These results are included in the analysis, and were assigned to the closest 9-month interval to the date of patient's termination in the study.

### 2.1. Dietary Assessment

To measure the effects of supplemental polyunsaturated fats upon lipids, it is necessary to measure the impact, if any, of the background diet. Because a single 24-HR cannot assess day-to-day variation in dietary intake [27], 3 unannounced 24-HRs were conducted on randomly selected days within a 3-week period (two weekdays and one weekend) at baseline and 18 months or time of the final visit. The dietary assessments, including reported intake of supplemental non-study marine and botanical oils or phytosterols, were completed utilizing a computer-assisted telephone interview with a multiple pass technique [28]. The 24-HR dietary recalls were administered by non-intervention registered dietitians, blinded to the patients' treatment group, and trained to collect dietary data using our interview system. The 24-HR-derived data were analyzed using the University of Minnesota Nutrition Coordinating Center Nutrition Data System for Research software (annually updated, current version: NDS-R 2009). Limitations to 24-HR dietary assessment in this population include factors related to self report, including possible underreporting of nutrient intake which has been observed in several studies [29].

### 2.2. Laboratory Measurement Methodology

Total cholesterol (TC) and triglyceride (TG) values were measured by conventional enzymatic methods. Briefly, cholesterol esters are converted to a colored quinine imine product. For HDL cholesterol, the lipoprotein particles are solubilized and release HDL cholesterol to react with cholesterol esterase and oxidase in the presence of chromogens to produce a color product. LDL cholesterol is calculated [30] according to the Friedewald Calculation which is TC-HDL − (TG ÷ 5) = LDL. Atherogenic index of plasma (AIP) was used to measure the risk of hypertension, diabetes, and vascular events in this population. The calculation of AIP is log 10 triglyceride/high-density lipoprotein cholesterol [31].

### 2.3. Statistical Methods

The three treatment arms were characterized at baseline using frequencies for categorical variables and means and standard deviations for continuous variables. Differences in mean values at 18 months or at the final visit were assessed for change in diet by the Student *t*-test [23]. Outcomes that were not normally distributed were log transformed for calculation of *P* values. Nontransformed data are reported for changes from baseline. To assess the effect of the intervention on lipids, lipid values were modeled using linear mixed modeling as a function of time (baseline, 9 month and 18 month, or the final visit treated as a categorical variable to allow for nonlinear trajectories), treatment arm, and their interaction, with adjustment for baseline value. To assess the overall changes over time, outcome measures were modeled using linear mixed modeling as a function of time, treatment arm, and with adjustment for baseline value. All analyses were intention to treat. Analyses included all participants with a baseline lipid measure.

## 3. Results

One hundred fifty-six patients were randomized, 56 received fish oil, 53 received borage seed oil, and 47 received both fish and borage seed oils. Patients were stratified by site (thus randomized to group within each site), and all sites were combined, resulting in non-significant differences per group. Serum lipids were obtained in 146 patients (93.6%) at baseline, 84 patients (53.8%) at 9 months, and 69 patients (44.2%) at 18 months.

Another 34 patients were screened but not randomized for the following reasons: arthritis medicine dose was not stable, too few tender joints, anticoagulated, high fish oil intake, high borage seed oil intake, medical issues, or abnormal laboratory values. The overall drop-out rate was 51% and was similar across groups: 25 in the borage oil group, 28 in the fish oil group, and 22 in the combination group. Reasons for dropout were mainly gastrointestinal distress (belching, bloating, diarrhea, nausea, cramping) or an inability to swallow the large number of rather sizable capsules. 

Patient characteristics at baseline are presented in Table 1. The mean age of participants was 59 years and the sample predominantly female (80%). Most were white (90%), married (69%), and had a mean body mass index (BMI) of 30.5. An equal number were retired (33%) or working full time (34%), and 16% listed themselves as disabled. There were no significant differences between groups. 

### 3.1. Diet

No significant change in dietary intake of fatty acids was seen (Table 2). However, a significant (*P* < 0.001) reduction in sources of dietary calcium (~200 mg) and a slight increase (1% of total calories) in protein intake were observed.

### 3.2. Weight

No significant differences in weight between groups were observed. Analysis was done using log-transformed weight; however, results are from the original scale. The mean increase when all groups were combined across the entire study was not significant: 0.5 lb increase at 9 months and 0.4 lb increase at 18 months (Table 4).

### 3.3. Lipids

There were no significant differences between groups for any lipid measure, with the exception of triglycerides. Therefore, all groups were, combined to evaluate the influence of marine and botanical oils on serum lipids (Table 3(a)). Lipids were done at baseline, 9 months, 18 months, or when the patient terminated the trial.

*Total cholesterol* reduction from baseline was 3.4 mg/dL (*P* = 0.129) at 9 months and 8.4 mg/dL (*P* ≤ 0.001) at 18 months. *LDL* was reduced significantly by 4.4 mg/dL at 9 months (*P* = 0.019) and 9.4 mg/dL at 18 months (*P* ≤ 0.001). *HDL *was significant: 4.0 mg/dL increase at 9 months (*P* ≤ 0.001) and 5.0 mg/dL increase at 18 months (*P* < 0.001). *TC/HDL ratio* decreased significantly over the time of the trial: a 0.26 reduction at 9 months (*P* < 0.001) and a reduction of 0.43 at 18 months (*P* < 0.001). *Triglycerides* were log transformed for analysis and reporting of *P* values. However, the coefficients and the differences reported are from the nonlog-transformed scale. Reductions in triglyceride concentrations were observed in all 3 groups (Table 3(a)). The overall decrease across the study period was 22.0 mg/dL at 9 months (*P* < 0.001) and 24.4 mg/dL at 18 months (*P* < 0.001). The TG reduction in the group treated with both oils was significantly greater (*P* < 0.031) than the borage oil or fish oil groups at 9 months, a pattern that persisted at 18 months (Table 3(b)). *Atherogenic index of plasma (AIP)* was reduced in all 3 groups (Table 3(a)). The overall decrease across the study period was 0.22 at 9 months (*P* < 0.001) and 0.26 at 18 months (*P* < 0.001). The reduction in the AIP was significantly greater (*P* = 0.011) at 9 months and 18 months in the group treated with both oils than that in the groups treated with either oil alone (Table 3(b)).

### 3.4. Sensitivity Analysis of Lipids

A sensitivity analysis was run to detect if missing data might have affected the study results. Missing data were imputed by substituting the baseline value. Since this would be the worst case scenario, in which all missing data return to baseline; analyses were repeated with the imputed data. The intragroup differences seen in triglyceride concentrations were not sustained with the imputed data. However, the intragroup differences seen with the AIP did persist. The significant change seen with all groups combined was also sustained with the imputed data, which indicates that missing data would not have a large impact on results from the all groups combined analyses.

### 3.5. Blood Pressure

Significant changes in blood pressure within and among groups were not observed. Systolic blood pressure increased 1.8 mm hg at 9 months and decreased 0.2 mm hg at 18 months. Diastolic blood pressure increased 2.3 mm hg at 9 months and 1.9 mm hg at 18 months (Table 4).

### 3.6. Erythrocyte Sedimentation Rate (ESR) [32] and C-Reactive Protein (CRP)

ESR is a common hematology test that is a nonspecific measure of inflammation. CRP is a protein found in the blood, and its levels increase in response to inflammation.

No significant differences in ESR or in CRP were seen among groups. However, when patients from all treatment groups were analyzed together, a modest but significant reduction in ESR was seen at 9 months, and ESR was still reduced from baseline at 18 months. A similar small but significant reduction in CRP was seen at 9 months, but not maintained at 18 months (Table 4).

## 4. Discussion

Part of the intrigue of research is the often unanticipated findings encountered. The current study was not designed to detect differences in lipids in patients with RA; hence, we lack a control group. Because marine and botanical oils given individually reduce joint inflammation in RA patients [14–18], and because the groups in this study showed improvement in the lipid profile, a trial of these oils with a placebo arm is warranted.

RA is a chronic systemic inflammatory disease. Mediators of inflammation and prothrombotic factors contribute to endothelial dysfunction and development of cardiovascular disease in RA patients [33]. There is little evidence that therapy for inflammation also leads to cardiovascular risk reduction in this group. Marine and botanical oils represent an excellent primary or secondary therapy for improvement of cardiovascular risk management in patients with rheumatoid arthritis.

Results of studies presented in this paper indicate that a GLA-enriched botanical oil (borage seed oil), an EPA/DHA-enriched fish oil, or a combination of these oils are useful for correcting dyslipidemia in patients with RA. Since there were no differences observed between the groups, with the notable exception of triglycerides and the AIP (shown separately in Table 3(b)), all 3 treatment groups were analyzed as a single group. Although lipid profiles of most patients were acceptable at baseline, patients taking these oils exhibit significant additional reductions in total and LDL cholesterol, triglycerides, the TC/HDL ratio, and the atherogenic index, and experience a significant increase in HDL cholesterol. All of these improvements in the lipid profile were seen after 9 months of therapy and increased after 18 months of oils administration. Particularly noteworthy is the group treated with both oils, as they experienced a significantly greater reduction in serum triglyceride concentrations and in the AIP than the groups on either oil alone. Oils enriched in GLA affect inflammation differently than oils enriched in EPA/DHA, and the anti-inflammatory and joint protective effects of the combination of these oils are synergistic in animal models [23]. Thus, it is possible that these different oils influence different aspects of TG synthesis or metabolism. Indeed, fish and botanical oils that provide EPA both reduce hepatic synthesis of TG in rats [34]. In humans the delta-5-desaturase that converts DGLA to arachidonic acid (AA) is sluggish, and we have not seen increases in circulating arachidonic acid after administration of GLA for 24 weeks [17]. Nonetheless, the possibility of increased circulating AA must be considered if treatment is to be long term. When fish oil is administered with borage oil to healthy individuals, bioconversion of GLA to AA is prevented [34], perhaps another reason for administering both GLA- and EPA-rich oils together. 

 All treatments were safe. Rates and types of adverse events were similar across all treatment groups, and as anticipated, were due entirely to mild to moderate gastrointestinal distress. The main reason for the large drop-out rate (in excess of 45%) was the large size and the number of capsules ingested each day over the 18-month-study period. It is possible to deliver much larger amounts of the individual polyunsaturated fatty acids (GLA, EPA, and DHA) in far smaller capsules than are needed to accommodate the natural marine and botanical oils, a strategy which should substantially reduce the dropout rate. 

 Alterations in diet can influence serum lipid concentrations [35]. However, the patients in our study did not change their diets over the course of the trial, suggesting that the improvements in their lipid profiles, including the significant increase in HDL cholesterol, are due to administration of the study oils. Most pharmacological treatments of dyslipidemia address reduction of LDL cholesterol [36, 37]. Since improvement in HDL cholesterol depends to a large extent on an exercise regimen [13, 38], many patients with RA are denied this manner of therapy. Thus, treatment with one or a combination of these oils could aid in the reduction of cardiovascular risk in RA patients whose disability impairs or prevents a prescribed exercise program.

Although LDL-C is the primary target of lipid-lowering therapy, other measures of the lipoprotein lipid profile, as reflected in the AIP and the TC/HDL-C ratios, are also associated with CVD risk. The AIP is a useful monitor of the lipid profile and its subsequent impact on the progression of cardiovascular risk [39]. In the study presented here, the AIP is significantly and beneficially altered at both 9 and 18 months compared to baseline. Patients in this study also exhibit a significant reduction in the TC/HDL ratio at 9 and 18 months, another indication of reduced CVD risk [40]. The safety of marine and botanical oils, and their remarkable impact observed in this study on the lipid profile of RA patients who are at increased risk for dyslipidemia and cardiovascular disease [1], suggest that these oils should have a prominent role in therapy of patients with RA.

 Additionally, there is some evidence [41] that these oils can substitute for treatment of RA patients with nonsteroidal anti-inflammatory drugs (NSAIDs). The adverse gastrointestinal and renal events associated with NSAID therapy are well known [42]. In addition, macrophages treated with a cyclooxygenase inhibitor in vitro exhibit greater vulnerability to formation of foam cells, a key element in development of atheromatous plaques [43]. Neither borage oil nor fish oil is associated with serious gastrointestinal events (ulceration, bleeding, perforation). In addition, whereas NSAIDs increase the incidence of myocardial infarction and stroke [44], fish oil reduces the risk of cardiovascular events in patients at risk, including those with RA [45]. The efficacy of omega-3-fatty acids in reducing mortality after a myocardial infarction [46] is further reason to recommend their use in patients with RA. Although studies in humans of the influence of GLA on serum lipids are scant, GLA administration appears to prevent increases in TC and LDL-C [47].

## 5. Conclusion

Chronic inflammation, experienced by patients with RA, includes development of microthrombi in small vessels and production of inflammatory cytokines and is associated with accelerated atherosclerosis [48]. The capacity of both GLA- and EPA-rich oils to reduce platelet aggregation [49] and production of inflammatory cytokines [18] and the ability of EPA to form resolvins and protectins, compounds that facilitate resolution of inflammation [50], further suggest their potential long-term therapeutic value in patients with RA. The current study suggests their beneficial effect on cardiovascular risk factors in patients with RA. Additional studies of marine and botanical polyunsaturated fatty acids—in isolated form in order to reduce the number and the size of capsules administered—are warranted to further determine their influence on lipid dysfunction in patients with RA and other diseases characterized by chronic inflammation. 

## Figures and Tables

**Table 1:** Baseline characteristics in patients with rheumatoid arthritis.

	Total (*N* = 146)*	
	Mean	SD

Age	59.24	11.58
BMI (kg/m^2^)	30.55	8.3

	N	%

Gender		
Male	28	19.2%
Female	118	80.8%

Marital status		
Married	100	69.0%
Other	45	31.0%

Race-collapsed		
White	132	90.4%
Minority	14	9.6%

Work status		
Full time	51	34.90%
Part time	14	9.60%
Other	81	55.50%

*None of the demographics are significantly different across the groups at baseline.

**Table 2:** Change in dietary factors from baseline to 18 months.

Change in	Mean	95% CI
Energy	9.01	−121.17 to 139.19
Total dietary fiber	−0.15	−1.75 to 1.45
Soluble dietary fiber	−0.15	−0.64 to 0.33
Insoluble dietary fiber	−0.05	−1.27 to 1.17
Calcium	−204.48	−375.78 to −33.19*
PUFA 18:3 (linolenic acid)	−0.03	−0.22 to 0.16
% calories from fat	−0.31	−2.28 to 1.65
% calories from SFA	0.09	−0.89 to 1.08
% calories from MUFA	−0.13	−0.91 to 0.66
% calories from PUFA	−0.26	−1.21 to 0.69
Omega-3-fatty Acids	0.13	−0.12 to 0.38
% calories from carbohydrate	−1.34	−3.19 to 0.51
% calories from protein	1.17	0.13 to 2.21*

*Values are presented as regression coefficient (95% CI) unless stated otherwise and control from baseline values. *P* < 0.001.

**Table:** (a) Serum Lipids and Atherogenic Index of Plasma

	Baseline mean (SD) (*N* = 145)	Change from baseline to 9 months (*N* = 83)	Change from baseline to 18 months (*N* = 69)
Total cholesterol	195.77 (37.48)	−3.45 (−7.88 to 0.98)	−8.43* (−12.99 to −3.86)
LDL	114.63 (32.20)	−4.39** (−8.03 to −0.74)	−9.43* (−13.75 to −5.11)
HDL	54.14 (16.21)	3.96* (2.44 to 5.49)	5.02* (3.24 to 6.81)
TC/HDL ratio^†^	3.83 (1.03)	−0.26* (−0.41 to −0.12)	−0.43* (−0.58 to −0.28)
Triglyceride^†^	138.05 (79.65)	−21.96* (−30.52 to −13.40)	−24.42* (−33.22 to −15.61)
Atherogenic index of plasma	0.84 (0.67)	−0.22* (−0.29 to −0.16)	−0.26* (−0.33 to −0.19)

Values are presented as regression coefficient (95% CI) unless stated otherwise and control from baseline values.  *P < 0.001  **P ≤ 0.05 ^†^ *P* value are from log-transformed data.

**Table:** (b) Triglycerides and atherogenic index of plasma (AIP) by group

	Combination group	Fish oil group	Borage oil group	*P* value
Triglyceride**				0.031
9 months	−30.81 (−46.58 to −15.03)	−20.50 (−35.16 to −5.85)	−16.57 (−30.86 to −2.27)	
18 months	−38.24 (−54.28 to −22.19)	−15.27 (−29.81 to −0.74)	−22.10 (−37.32 to −6.89)	

AIP				0.011
9 months	−0.33 (−0.45 to −0.20)	−0.20 (−0.32 to −0.08)	−0.17 (−0.33 to 0.002)	
18 months	−0.45 (−0.57 to −0.32)	−0.16 (−0.28 to −0.05)	−0.21 (−0.33 to −0.09)	

**P value is from log transformation. Changes shown are from the original scale for the group × time interaction and control for baseline values. Values are presented as regression coefficient (95% CI).

**Table 4:** Change from baseline for anthropometric and inflammatory markers.

	9 months (*N* = 88)	18 months (*N* = 71)
Weight^†^	0.52 (−1.41 to 2.46)	0.35 (−2.43 to 3.14)
Systolic blood pressure	1.77 (−0.66 to 4.20)	−0.24 (−3.37 to 2.90)
Diastolic blood pressure	2.32* (0.56 to 4.09)	1.88** (−0.003 to 3.79)
ESR^†^	−5.39* (−9.71 to −1.07)	−4.42** (−9.22 to 0.38)
CRP^†^	−0.65* (−1.20 to −0.10)	−0.09 (−0.77 to 0.60)

*P < 0.001.

**P ≤ 0.05.

^†^ *P* values are from log-transformed data.

*N* = 90 at 9 months and *N* = 72 at 18 months for blood pressure measurements.

*N* = 81 at 9 months and *N* = 67 at 18 months for ESR.

*N* = 66 at 9 months and *N* = 58 at 18 months for CRP.
